# Does Incipient Dementia Explain Normal Cognitive Decline Determinants? Lothian Birth Cohort 1921

**DOI:** 10.1037/pag0000241

**Published:** 2018-05-10

**Authors:** Ruth A. Sibbett, Tom C. Russ, Alison Pattie, John M. Starr, Ian J. Deary

**Affiliations:** 1 Alzheimer Scotland Dementia Research Centre and Centre for Cognitive Ageing and Cognitive Epidemiology, University of Edinburgh; 2 Centre for Cognitive Ageing and Cognitive Epidemiology, University of Edinburgh; 3 Alzheimer Scotland Dementia Research Centre and Centre for Cognitive Ageing and Cognitive Epidemiology, University of Edinburgh

**Keywords:** dementia, cognitive ageing, cognitive ability, risk factors, cognitive decline

## Abstract

The presence of an apolipoprotein E (*APOE*) ε4 allele, lower physical fitness, smoking, and lower serum vitamin B-12 have been reported as contributing to poorer cognitive function in LBC1921 at age 79, after adjusting for childhood intelligence. Because incident dementia was not previously ascertained within LBC1921, it is possible that preclinical or unrecognized cases at age 79 influenced findings. Dementia cases arising over approximately 16 years of follow-up were determined by a consensus using evidence from electronic medical records, death certificates, and clinical reviews. The analyses from the original reports were repeated after the exclusion of those who had developed dementia. In a subsequent set of analyses, the authors considered the potential impact of terminal decline, excluding those participants who died within 4 years of baseline testing. Positive *APOE* ε4 status was found to be associated with poorer Logical Memory (Wechsler, 1987) at age 79 (*F*(1, 355) = 8.16, *p* = .005, η_p_^2^ = 0.022; *n* = 359) and lower Moray House Test (Scottish Council for Research in Education, 1933) score at age 79 (*F*(1, 357) = 4.27, *p* = .04, η_p_^2^ = 0.012; *n* = 363). Lower age 79 IQ was associated with smoking (*F*(2, 360) = 3.67, *p* = .026, η_p_^2^ = 0.020; *n* = 367), lower vitamin B-12 (*S*β = 0.11, *p* = .014; *n* = 367), and poorer physical fitness (*S*β = 0.21, *p* < .001; *n* = 359). Only the relationship with physical fitness remained significant after excluding those who died within 4 years of baseline (*S*β = 0.203, *p* < .001; *n* = 310). Unrecognized dementia had little or no effect on determinants of lifetime cognitive ageing in LBC1921. Terminal decline may have accounted for the associations with age 11 to age 79 cognitive change.

Cognitive function in older age is a critical factor in maintaining independence and well-being (Fillit et al., 2002). Some aspects of cognitive ability are known to decline with advancing age, even in the absence of dementia (Robert S. Wilson et al., 2002). As the global population ages, it is therefore increasingly important to understand the determinants of differences in normal cognitive ageing. Furthermore, with an improved understanding of normal cognitive ageing it might be possible to distinguish it more clearly from pathological ageing. This will become increasingly important as the diagnosis of neurodegenerative conditions shifts earlier and earlier to prodromal and preclinical states. Evidence for the association between many different factors and nonpathological cognitive ageing are documented within the literature. A 2010 systematic review highlighted smoking and the apolipoprotein E (*APOE*) ε4 genotype as risk factors for greater cognitive decline, whereas better physical health was identified as a protective factor (Plassman, Williams, Burke, Holsinger, & Benjamin, 2010).

The interpretation of findings relating to normal cognitive ageing is, however, often limited by the difficulty in distinguishing whether observed associations might be explained by the presence of the early stages of neurodegeneration (preclinical or prodromal Alzheimer’s disease, for instance) in some participants. This is particularly important when investigating factors such as depression and impaired physical fitness that might themselves be early symptoms of neurodegenerative disease (Plassman et al., 2010). Articles describing normal cognitive ageing typically exclude participants with known or suspected dementia at baseline or at the time of analysis. In most cases, studies perform dementia ascertainment in parallel with monitoring cognitive change, either using a planned dementia assessment protocol or recording the diagnosis as an incidental finding (Packard et al., 2007). A lag period between collecting the cognitive function results and determining dementia status is, however, necessary to reduce the number of incipient cases missed. Studies that retrospectively exclude participants who went on to develop dementia during an extended period of follow-up are rarer (Bretsky, Guralnik, Launer, Albert, & Seeman, 2003; Feng et al., 2012; Fillenbaum et al., 2001; Fitzpatrick et al., 2007; Praetorius, Thorvaldsson, Hassing, & Johansson, 2013; Small, Dixon, McArdle, & Grimm, 2012; Yaffe et al., 1999). Previous prospective studies have confirmed a subtle cognitive decline in nondemented participants of older-age, who went on to develop dementia (Bäckman, Jones, Berger, Laukka, & Small, 2005; Lange et al., 2002). A meta-analysis of previous studies has shown that *APOE* ε4 is significantly associated with adverse effects on a number of domains of cognitive function in nondemented older-adults (Wisdom, Callahan, & Hawkins, 2011). *APOE* ε4 carriers performed significantly poorer on tests of episodic memory (*d* = −0.14 (−0.21, −0.07), *p* < .01), global cognitive ability (*d* = −0.05 (−0.10, −004), *p* < .05), executive functioning (*d* = −0.06 (−0.12, −0.004), *p* < .05), and perceptual speed (*d* = −0.07 (−0.13, −0.01), *p* < .05; Wisdom et al., 2011). One of the studies included in the meta-analysis did, however, indicate that the association between *APOE* ε4 and cognitive function did not remain when those with preclinical dementia were excluded from the sample (Bondi, Salmon, Galasko, Thomas, & Thal, 1999; Lange et al., 2002). Such studies highlight the importance of evaluating the impact of preclinical dementia when investigating the determinants of nonpathological cognitive ageing. Like most studies of their kind, early studies of possible determinants of normal cognitive ageing between age 11 and age 79 in the Lothian Birth Cohort, 1921 (LBC1921) did not have a follow-up period in which to determine incident dementia. We address and correct this limitation in the present report, providing further evidence regarding the potential effect of preclinical dementia on studies of nonpathological cognitive ageing.

LBC1921 is a narrow-age cohort (*N* = 550), mostly recruited from the City of Edinburgh and its surrounding area. Most participants had taken part in a Scottish national intelligence test aged 11 years (Deary, Gow, Pattie, & Starr, 2012). Participants were recruited at a mean age of 79 years and have been followed-up into their 90s. Studies of this cohort have reported four factors associated with almost-lifetime cognitive ageing, from age 11 to age 79 years (i.e., factors associated with cognitive function at age 79 years after adjusting for childhood IQ): smoking, lower physical fitness, *APOE* ε4 status, and vitamin B-12 levels. Smoking was associated with greater relative cognitive decline from age 11 to age 79 within the LBC1921 (Deary et al., 2003). A lower level of overall physical fitness at 79 years was associated with less successful cognitive ageing (Deary, Whalley, Batty, & Starr, 2006). Specifically, a higher mental test score at age 79, after adjustment for intelligence at age 11, was correlated with increased FEV_1_ (forced expiratory volume in 1 second; a measure of lung function), decreased 6-metre walk time, and increased grip strength after adjusting for intelligence at age 11 (Deary et al., 2006). Possessing an *APOE* ε4 allele was associated with both poorer Logical Memory Test scores at age 79, and cognitive decline from age 11 to age 79 in this cohort (Deary, Whiteman, Pattie, & Starr, 2004; Deary et al., 2002). Lower serum vitamin B-12 levels at age 79 was associated with greater relative cognitive decline from age 11 to age 79 (Starr, Pattie, Whiteman, Deary, & Whalley, 2005).

In these previous studies, LBC1921 was treated as a homogeneous group with regard to cognitive ageing, but the cohort might have contained at least two separate groups: that is, one group with “normal” or nonpathological cognitive ageing, and another group who are subject to accelerated cognitive change because of a pathological process, most likely dementia. This is of particular importance given the recognized associations between *APOE* ε4, smoking, physical fitness, and dementia, in addition to the associations with cognitive ageing mentioned above. A systematic review and Delphi consensus study published in 2014 concluded that both smoking and physical inactivity were important modifiable risk factors for dementia (Deckers et al., 2015). The review included a meta-analysis of eight studies that found that current smoking was associated with a 59% increase in risk for Alzheimer’s disease (Deckers et al., 2015; Peters et al., 2008). Further to the evidence linking physical inactivity and dementia, there is evidence within the literature specifically linking poorer grip strength, lung function, and walking speed with dementia (Camargo et al., 2016; Yoon et al., 2015). The oldest in the population are less well represented in dementia research and as such, the majority of the evidence for smoking and physical inactivity as risk factors for dementia is taken from studies involving participants in either earlier old age or from a wide age range. Some studies have refuted the importance of these risk factors with advancing age (Piguet et al., 2003; Verghese et al., 2003; Wang, Fratiglioni, Frisoni, Viitanen, & Winblad, 1999), but the paucity of studies investigating such factors in the oldest-old mean that it is not possible to conclusively rule out the possibility that these risk factors had an effect on the findings of the original articles considered in this study. Possession of an *APOE* ε4 allele has been shown to increase the risk of dementia, and Alzheimer’s dementia in particular (Corder et al., 1993). While the potency of *APOE* ε4 as a risk factor has been shown to reduce in oldest-age, the same meta-analysis confirms that it continues to increase the risk for dementia in oldest-age cohorts (Farrer et al., 1997). The presence of at least one *APOE* ε4 allele has also been shown to increase the risk for dementia after age 79 in LBC1921 (Sibbett, Russ, Deary, & Starr, 2017b). Therefore, it remains an important consideration in this study. The relationship between vitamin B-12 and dementia is less clear, with studies demonstrating inconsistent findings (Agnew-Blais et al., 2015). A 2012 systematic review found no association between serum vitamin B-12 levels and risk of dementia, but did demonstrate an association between poor vitamin B-12 status and increased risk of dementia in studies using alternative biomarkers of vitamin B-12 status (holotranscobalamin and methylmalonic acid; O’Leary, Allman-Farinelli, & Samman, 2012). Low levels of serum B-12 and elevated total homocysteine—that may be caused by vitamin B-12 deficiency—have been linked with increased risk of dementia in the oldest-old (Kivipelto et al., 2009; Wang et al., 2001). Notwithstanding the inconsistencies between studies, and in particular between studies of early old age and the oldest-old, it is important to test whether the apparent associations of vitamin B-12, physical fitness, smoking, and *APOE* ε4 genotype with nonpathological cognitive ageing in LBC1921 were driven by a subgroup who subsequently developed dementia.

The LBC1921 findings listed above were based on analyses conducted soon after the sample was recruited aged around 79 years. At that time, people with possible dementia were excluded if they scored <24 on the MMSE or reported a dementia diagnosis at baseline. After the participants had been followed up for approximately 16 more years, it was possible to ascertain incident dementia cases and to repeat the previous analyses excluding people who subsequently developed dementia to isolate any group with true “normal” cognitive ageing.

In addition to performing these sensitivity analyses, we also planned to use the available follow-up data to investigate the possible impact of so-called “terminal decline” on the original findings. Cognitive decline has been found to accelerate in the years before death, with one particular study demonstrating accelerated cognitive decline 43 months from death (R. S. Wilson, Beckett, Bienias, Evans, & Bennett, 2003). The original LBC1921 findings that are reconsidered in this article were produced shortly after recruitment, and any effect of terminal decline was not, therefore, examined. To investigate the possible role of terminal decline we, therefore, repeat the previously reported analyses after excluding those eligible participants who died within 4 years of baseline testing at 79 years.

## Method

### Participants

Participants were members of the LBC1921. Most had taken part in the Scottish Mental Survey of 1932 (SMS1932), completing a validated test of general intelligence, the Moray House Test No. 12, at age 11 years. The LBC1921 is described in detail elsewhere, and, at recruitment, comprised 550 community-dwelling, generally healthy older people, mostly from the Lothian area of Scotland, who were recruited to follow-up at mean age 79.1 years (*SD*: 0.6; Wave 1; Deary et al., 2012). Surviving participants underwent four subsequent waves of follow up at mean ages of 83, 87, 90, and 92 years (Waves 2 to 5). Study data included measures of sociodemographic, psychological, cognitive, medical, physiological, and genetic factors, collected by questionnaire and clinical testing. Dates and causes of death were supplied prospectively by the National Records of Scotland (previously General Registrar’s Office, Scotland). The Lothian Research Ethics Committee (test Waves 1–3) and the Scotland A Research Ethics Committee (test Waves 4–5) provided ethical approval for the studies. From Wave 4, participants provided written consent for data linkage and access to health records. In line with the previous cognitive ageing articles, only participants with an age-79 Mini-Mental State Examination (MMSE; Folstein, Folstein, & McHugh, 1975) score of 24 or higher and no self-reported history of dementia at Wave 1 were included in these sensitivity analyses (Deary et al., 2003, 2004, 2006; Starr et al., 2005). We considered repeating our analyses using a stricter MMSE cut-off to identify those who might subsequently develop dementia, but a receiver operator characteristic (ROC) curve determined that the discriminating power of the MMSE was insufficient to determine future dementia outcomes (area under curve = 0.54) in this cohort, and it was in fact little better than random allocation. It is possible that the variation in cognitive reserve between individuals limits the use of the MMSE as a predictive tool.

### Dementia Ascertainment

Dementia ascertainment methodology for this cohort has been described previously (Sibbett et al., 2017b), and will be outlined briefly here. Evidence for subsequent dementia or cognitive impairment after recruitment was collected from death records, and medical and psychiatric electronic records. For a small number of participants, additional information was available as a result of clinical assessments, performed in the research or NHS setting by the authors (JMS and TCR). Data were collected up to June 2016, when participants were aged approximately 95 years. Each case with any evidence suggestive of dementia or cognitive decline was considered at a consensus meeting that included both a geriatrician and a psychiatrist. The meeting agreed whether the evidence supported a diagnosis of dementia. Dementia cases were determined to be possible or probable cases according to a standard set of criteria (see Table 1). For the purposes of these sensitivity analyses, both probable and possible cases were considered dementia cases and, therefore, excluded from the main analyses.[Table-anchor tbl1]

### Cognitive Testing

Participants took a validated test of general mental ability (the *Moray House Test (MHT) No. 12*) at age 11 and age 79 (Scottish Council for Research in Education, 1933). The test consisted of 75 items, completed over 45 min and the maximum achievable score was 76. The MHT scores were corrected for age (in days) and converted to IQ-type scores (*Mean* = 100, *SD* = 15). At age 79, an additional battery of cognitive tests was administered to assess some major domains of cognitive function. Verbal declarative memory was assessed using the Logical Memory subtest from the Wechsler Memory Scale-Revised (Wechsler, 1987). Participants were read a short story (story A) containing 25 memory items and immediately after this they were asked to recall as much as possible. This was repeated for a second story (story B). After a delay of approximately 30 min, participants were asked to recall as much detail as possible from both stories. Immediate and delayed test scores were summed to form a single score ranging from 0 to 100. The phonemic Verbal Fluency test (Lezak, 1995) was used as a measure of one facet of executive function. Participants were required to name as many words as possible beginning with the letter C in 1 min. This process was repeated for the letters F and L and the total number of correct words given is the overall test score. Raven’s Standard Progressive Matrices (Raven & Court Jr, 1977) was used as a measure of nonverbal or abstract reasoning. The test comprised 60 items, with each item representing a pattern that required completion. The score was given by the number of items completed correctly within the 20-minute time limit.

### Exposures Associated With Cognitive Decline in Previous Studies

LBC1921 participants provided samples of venous blood at baseline, aged 79. Venous blood was used for DNA extraction. *APOE* ε4 status was determined by polymerase chain reaction (PCR) amplification of a 227 base pair fragment of the *APOE* gene containing two polymorphic sites that account for three alleles: ε2, ε3, and ε4 (Wenham, Price, & Blundell, 1991). These alleles were distinguished by restriction digest with the enzyme *Cfo1*, followed by electrophoresis in 4% NuSieve gel. Venous blood samples collected at a clinical research facility were used to measure serum vitamin B-12.

Grip strength in the dominant hand was measured using a Jamar Hydraulic Hand Dynamometer, and the best of three trials was used. Lung function was recorded as forced expiratory volume in 1 s (FEV_1_), measured using a microspirometer; the best of three attempts was recorded. The time taken to walk 6 m at a normal pace was recorded. Grip strength, FEV_1_, and 6-m walk-time were correlated and were combined using principal component analysis to obtain a summary fitness trait (we use the term trait, though strictly speaking this is a score from a first unrotated component). Smoking status was reported by participants, and coded as never-, ex-, or current-smoker. Time from enrolment to death was calculated by subtracting the age in days at baseline testing from the age in days at death.

### Statistical Analyses

For each of the original cognitive ageing reports (Deary et al., 2002, 2003, 2004, 2006; Starr et al., 2005), the most significant findings were selected to be investigated in these sensitivity analyses. We included two articles that investigated the effect of *APOE* ε4 on cognitive ageing (Deary et al., 2002, 2004). The first (named Article 1 hereafter) demonstrated that possessing an *APOE* ε4 allele was significantly associated with greater cognitive decline from age 11 to age 79 using the same test of mental ability at both ages (Deary et al., 2002). In the second *APOE* ε4 article (named Article 2 hereafter), the main finding was that the presence of at least one *APOE* ε4 allele contributed significantly to lower Logical Memory test scores at age 79 (Deary et al., 2004). It was important to consider both articles given the relevance of memory decline in dementia and general cognitive decline.

Current smoking was significantly associated with a lower score on age 79 IQ (age-adjusted MHT scores at age 79, though it was stated in the report as age 80) compared with ex-smokers and never-smokers (Deary et al., 2003). Lower serum vitamin B-12 at age 79 was associated with greater relative cognitive decline between age 11 and 79 (Starr et al., 2005). The main finding from the physical fitness article was that lower general physical fitness component—derived from principal component analysis of three individual measures—was significantly associated with greater relative cognitive decline from age 11 to age 79 (Deary et al., 2006).

Each exposure (*APOE* ε4 status, smoking, vitamin B-12, and general physical fitness) was considered separately in the first instance; in each case, the analytical approach used repeated that of the previous reports. We then conducted analyses that included all four of these exposure variables simultaneously. The method and model for each analysis was as follows. *APOE* ε4 (Article 1): General linear modeling, with MHT score at age 79 (standardized as an IQ-type score) as the outcome or dependent variable, MHT score at age 11 (standardized as an IQ-type score) as covariate and sex and *APOE* ε4 carrier status as fixed factors; *APOE* ε4 (Article 2): General linear modeling, with Raven’s Standard Progressive Matrices, Logical Memory subtest, and Verbal Fluency test scores as dependent variables, sex and *APOE* ε4 status as fixed factors, and age 11 IQ as covariate; Smoking: General linear modeling, with age 79 IQ as the dependent variable, sex and smoking as fixed factors, and age 11 IQ as covariate; Vitamin B-12: Linear regression, with age 79 IQ as the dependent variable and vitamin B-12, age 11 IQ, sex, *APOE* ε4 status, smoking status, use of statins and number of prescribed drugs as independent variables; Physical fitness: Linear regression, with age 79 IQ as the dependent variable and sex, age 11 IQ, fitness trait, smoking status, *APOE* ε4 status and social class as independent variables; Combined analyses: (a) Linear regression including the four main variables simultaneously (*APOE* ε4 status, smoking status, vitamin B-12, and fitness trait), plus age 11 IQ and sex, with IQ at age 79 as the outcome variable; (b) Multivariate general linear modeling, with Raven’s Matrices, Verbal fluency and Logical Memory as dependent variables, *APOE* ε4 status, sex, and smoking status as fixed factors and fitness, vitamin B-12 and age 11 IQ as covariates. To improve the clarity of the effect size, we also present estimated marginal means (95% confidence interval [CI]) with related effect size (Cohen’s *d*) for categorical risk factors. For comparison, this is given alongside the same statistics for the study cohort when dementia cases were included. After each of the individual analyses, we completed a subsequent analysis in which participants with probable dementia, or no dementia were included. Forming a between-groups variable, we included an interaction term with the risk factor to determine whether it varied as a function of the inclusion group. Finally, we repeated the each of the main individual analyses after the exclusion of those participants who had died within 4 years of baseline testing. The methods and models were as shown above. Statistical analyses used IBM SPSS, Version 21.

## Results

There were 550 participants attended the baseline wave of data collection at age 79 years. For each analysis, we excluded 130 participants: 2 participants had reported a diagnosis of dementia at Wave 1; 9 participants scored less than 24 on the MMSE at Wave 1; 2 participants were missing MMSE scores at baseline; and there were 117 participants for whom we had ascertained a diagnosis of dementia in about 16 years of follow up, to age 95 years. Each analysis then repeated the other exclusion criteria and requirements as recorded in the original reports and, as a result, the number of participants with complete data for each analysis varied slightly between the separate outcomes. The numbers providing full data for each analysis were as follows: *APOE* ε4, (Article 1) *n* = 363 (Article 2) *n* = 359; smoking, *n* = 367; vitamin B-12, *n* = 367; and physical fitness, *n* = 359.

### *APOE* ε4

#### *APOE* ε4 Article 1: MHT score as outcome (Deary et al., 2002)

Of 363 participants with complete data, 210 (57.9%) were female and 79 (21.8%) possessed at least one *APOE* ε4 allele. On general linear modeling, a lower MHT score at age 11 years was associated with a lower MHT score at age 79 (*F*(1, 357) = 239.4, *p* < .001, η_p_^2^ = 0.401). Sex was also associated with MHT score at age 79 (*F*(1, 357) = 4.76, *p* = .03, η_p_^2^ = 0.013) as was the presence of an *APOE* ε4 allele (*F*(1, 357) = 4.27, *p* = .04, η_p_^2^ = 0.012; see Table 2). These results were consistent with those of the original study, which found a similar effect size for each of the three variables (see Table 1). The exclusion of dementia cases had very little impact on the effect size (*d* = −0.23 (−0.44, −0.03); *d* = −0.26 (−0.51, −0.01) and estimated marginal means (see Table 3). The complete results for each of the main sensitivity analyses and those for the combined analyses are shown in supplementary material File A, Tables S1–S7.[Table-anchor tbl2][Table-anchor tbl3]

#### *APOE* ε4 Article 2: Logical Memory, Raven’s Matrices, and verbal fluency as outcomes (Deary et al., 2004)

Of the 359 participants with full data for these analyses, 208 were female (57.9%) and 78 were carriers of at least one *APOE* ε4 allele (21.7%). On univariate analyses (*t* test) there was no significant difference between those with and without an *APOE* ε4 allele in age 11 IQ, *p* = .50, or age 79 MMSE, *p* = .48. On general linear modeling, positive *APOE* ε4 status was found to contribute to a lower Logical Memory test score at age 79 years (*F*(1, 355) = 8.16, *p* = .005, η_p_^2^ = 0.022), but not to Raven’s Matrices (*F*(1, 355) = 3.56, *p* = .06, η_p_^2^ = 0.010), or to Verbal Fluency (*F*(1, 355) = 26.52, *p* = .664, η_p_^2^ = 0.001) tests’ scores. The findings from this analysis replicate those found in the original article (see Table 2). In both the original and new analyses, age 11 IQ contributed significantly to all three measures whereas sex contributed to Raven’s Matrices only. The effect size (Cohen’s *d*) relating to Logical Memory test score remained relatively stable after the exclusion of those participants who went on to develop dementia (*d* = −0.35 (−0.56, −0.14); *d* = −0.36 (−0.61, −0.11); see Table 3).

### Smoking

Of the 367 participants with data available for these analyses, 211 were female (57.5%). There were 189 participants were ex-smokers (51.5%), 30 were current smokers (8.2%), and 148 were never smokers (40.3%). The mean age at starting smoking was 18.4 years (*SD*: 5.5) years (range of 7–60 years), and only four ever-smokers started before the age of 11. On general linear modeling, smoking was found to be associated with a lower IQ at age 79 years (*F*(2, 360) = 3.67, *p* = .026, η_p_^2^ = 0.020; see Table 2). Age 11 IQ was also found to be significant, whereas sex was not. As shown in Table 2, the original article also found a significant association between smoking and age 79 IQ. The effect size (Cohen’s *d*) was relatively unaffected by the exclusion of dementia cases when current smoking was compared with ex-smoking (*d* = −0.44 (−0.8, −0.08); *d* = −0.45 (−0.84, −0.06)) and similarly, when current smoking was compared with never smoking (*d* = −0.43 (−0.79, −0.06); *d* = −0.53 (−0.92, −0.13); see Table 3). IQ at age 79 was significantly lower for current smokers, compared with both ex-smokers (*p* = .024, *mean difference* = −5.0, 95% CI [−9.3 to −0.7]) and never-smokers (*p* = .007, *mean difference* = −6.2, 95% CI [−10.6 to −1.7]), and again, this replicated findings from the original article.

### Vitamin B-12

Of the 367 participants eligible for inclusion in these analyses, 211 were female (57.5%) and 326 had serum vitamin B-12 levels available (88.8%). The mean vitamin B-12 level was 388 (*SD* 162) ng/L. To prevent bias from participants with very high serum levels that were the result of treatment for vitamin B-12 deficiency, those with a serum level more than 3 *SD*s higher than the mean were excluded (*n* = 8). The resulting mean level for included cases was 374.0 (*SD* 134.5) ng/L. Serum vitamin B-12 levels were standardized and stored as *z* scores for the purposes of analyses. As in the original article, the linear regression results demonstrated a significant association between lower vitamin B-12 and lower age 79 IQ (*S*β = 0.124, *p* = .006, *R*^2^ change = 0.015). After adjusting for sex, *APOE* ε4 status, smoking status, use of statins, and number of prescribed drugs, vitamin B-12 continued to be associated significantly with age 79 IQ (*S*β = 0.110, *p* = .014, *R*^2^ change = 0.012; see Table 2). This was again the same outcome of the analysis in the original article (*S*β = 0.095, *p* = .011). We replicate the association between vitamin B-12 and age 79 IQ by repeating our analysis without excluding dementia cases (*S*β = 0.087, *p* = .022, *R*^2^ = 0.007).

### Physical Fitness

A total of 359 participants met the inclusion criteria for these analyses, of which 208 were female (57.9%). The sex and height adjusted fitness measures—grip strength, 6-m walk time, and FEV_1_— were all significantly correlated (*p* ≤ .01). Principal component analysis identified a single component that accounted for 48% of the total variance. The loadings on this first unrotated component—termed “fitness”—were as follows: grip strength = 0.75; 6-m walk time = −0.65; FEV_1_ = 0.68. In the present analyses, age 11 IQ was not significantly associated with FEV_1_ (0.097, *p* = .067) as it was in the original report (*p* = .03; Deary et al., 2006). IQ at age 79 correlated significantly with all three individual fitness measures (grip strength = 0.154, *p* = .003; 6-m walk time = −0.193, *p* < .001; FEV_1_ = 0.174, *p* = .001) and with the combined fitness trait (0.231, *p* < .001). Linear regression analyses showed that the variables contributing significantly (*p* ≤ .05) to variance in IQ scores at age 79 were: age 11 IQ (38.3% of variance); fitness (4.7%), sex (0.8%), and social class (1.8%). Fitness accounted for a higher percentage of variance in this analysis when compared with the original analysis (3.3%; see Table 2). When dementia cases were included, the following variables were found to contribute to age 79 IQ: age 11 IQ (42.4% of variance); fitness (3.1%), social class (1.2%), sex (0.7%), and *APOE* ε4 (0.6%). Smoking status did not demonstrate a significant contribution to variance in either analysis (*p* > .05).

We repeated each of the main analyses with the study sample comprising of participants with probable dementia, or no dementia. Dementia status was included as a between-groups variable and we included an interaction term with the risk factor to determine whether the effect varied as a function of the dementia group. The interactions between dementia and *APOE* ε4 status, dementia and smoking status, dementia and vitamin B-12 level, and dementia and fitness were not significantly associated with standardized MHT test score or IQ at age 79. The *APOE* ε4 carrier status by dementia status interaction was associated with Raven’s Matrices test score at age 79 (*p* = .006), but not Logical Memory or Verbal Fluency test scores. Further details of these results can be seen in supplementary material File B.

### Combined Analyses

Linear regression including the four main exposure variables (*APOE* ε4 status, smoking status, vitamin B-12, and fitness trait) showed that age 11 IQ (34.2%), fitness (5.6%), vitamin B-12 (2.1%), and sex (1.7%) contributed significantly (all *p* < .01) to variance in age 79 IQ. When dementia cases (*n* = 103) were included in the analysis, age 11 IQ (39.9%), fitness (3.4%), vitamin B-12 (1.1%), and sex (1.1%) continued to contribute significantly to variance (*p* < .01). *APOE* ε4 status and smoking did not enter into the models.

Multivariate general linear modeling showed positive *APOE* ε4 status to be associated with lower Logical Memory test scores at age 79 years (*F*(1, 301) = 5.5, *p* = .02, η_p_^2^ = 0.018). Lower levels of general physical fitness at age 79 years was associated with both lower Verbal Fluency (*F*(1, 301) = 12.3, *p* = .001, η_p_^2^ = 0.039) and lower Raven’s Matrices (*F*(1, 301) = 18.0, *p* < .001, η_p_^2^ = 0.056) test scores. When dementia cases (*n* = 103) were included in the same analysis, the same associations were found: *APOE* ε4 and Logical Memory (*F*(1, 392) = 5.5, *p* = .02, η_p_^2^ = 0.014); fitness and Verbal Fluency (*F*(1, 392) = 12.8, *p* < .001, η_p_^2^ = 0.032); fitness and Raven’s Matrices (*F*(1, 392) = 13.6, *p* < .001, η_p_^2^ = 0.034). In addition, lower vitamin B-12 was associated with lower Raven’s Matrices test scores (*F*(1, 392) = 5.6, *p* = .02, η_p_^2^ = 0.014). Smoking did not contribute significantly to any of the three outcomes.

### Terminal Decline

The number of participants who died within 4 years of baseline testing and were, therefore, excluded from the “terminal decline” analyses were as follows: *APOE* (Article 1), *n* = 52; *APOE* (Article 2), *n* = 50; smoking, *n* = 52; vitamin B-12, *n* = 52; fitness, *n* = 49. After the exclusion of these participants—and those who had developed dementia—the resulting cohort size for reanalyses ranged from *n* = 309–315. The main individual analyses were repeated and the association between fitness and age 79 IQ remained significant (*S*β = 0.203, *p* < .001). The results for *APOE* ε4 (Article 1: *F*(1, 306) = 1.30, *p* < .255, η_p_^2^ = 0.004; Article 2: *F*(1, 304) = 2.95, *p* = .087, η_p_^2^ = 0.010), smoking (*F*(2, 308) = 1.97, *p* = .141, η_p_^2^ = 0.013) and vitamin B-12 (*S*β = 0.087, *p* = .075) did not reach significance (see Table 2). The estimated marginal means and effect sizes (Cohen’s *d*) for the categorical variables are shown in Table 3. The complete results for the terminal decline analyses are shown in supplementary material File A, Tables S1–S5.

### Power Calculations

We completed post hoc power calculations to describe the statistical power of the new analyses, relative to the original analyses. The statistical power to detect an effect of the same magnitude at *p* < .05, as was observed for the complete study sample, was reduced after the exclusion of dementia cases in each analyses. The reduction in power ranged from 3% (from 0.99 to 0.96 for the physical fitness analysis) to 18% (0.60 to 0.49 for the smoking analysis). The statistical power was reduced further after the additional exclusion of those participants who died within 4 years of baseline testing; with reductions in power ranging between 6% (from 0.96 to 0.93 for physical fitness) and 28% (from 0.58 to 0.42 for *APOE* ε4 Article 1 and from 0.60 to 0.43 for the smoking analysis).

## Discussion

These sensitivity analyses, completed by repeating analyses conducted in five of our team’s previous reports after excluding participants who subsequently developed dementia, verified previous findings of LBC1921 studies. The presence of an *APOE* ε4 allele, smoking, lower physical fitness, and lower vitamin B-12 were all associated with greater relative cognitive decline between age 11 and 79 years even after excluding those who had developed dementia in the next 16 years. The effect sizes were similar in magnitude to the previous findings and, therefore, we can be more confident that prodromal or undiagnosed dementia had little influence on the original findings. However, our analyses did suggest that terminal decline could have influenced the results, with only physical fitness remaining significant after the exclusion of those who died within 4 years of baseline testing. With a smaller sample size, we had less power for these analyses and, therefore, we are cautious when considering the results.

### Comparison With Previous Literature

Like the original articles that investigated the relationship between *APOE* ε4 and cognitive ageing, we found that the presence an *APOE* ε4 allele contributed to poorer performance on a Logical Memory test at age 79 years, and contributed to general cognitive decline from age 11 to age 79. Using a robust method to exclude dementia cases we have minimized the possibility that dementia had caused some of the effect seen previously. Our findings agree with a previous meta-analysis, which found that *APOE* ε4 carriers performed poorer on tests of episodic memory, global cognitive ability, executive function and perceptual speed (Wisdom et al., 2011). The effect sizes for episodic memory and global cognitive ability were noted to increase with advancing age (Wisdom et al., 2011). Our results did not remain significant after accounting for possible terminal decline. Several studies have considered the effect of *APOE* ε4 on mortality and although a relationship is described, it is typically explained by the presence of dementia. If we accept our results as correct, despite the reduced sample size, it is possible that the participants who demonstrated a link between *APOE* ε4 and cognitive decline in our main analyses would in fact have gone on to develop dementia had they survived.

The findings for smoking and cognitive decline from the present analyses were consistent with the original article (Deary et al., 2003). Our findings also agree with the conclusions of other previous studies identifying smoking as a risk factor for cognitive decline (Yaffe et al., 2009), and recording an increased risk of decline in current smokers when compared with never-smokers and ex-smokers (Sabia et al., 2012). The potential for underestimating the effect of smoking on cognition as a result of higher rates of death and dropout among smokers is noted from the results of a previous study (Sabia et al., 2012). If risk of death is increased among smokers, this may explain why the relationship between smoking and cognition becomes nonsignificant after the exclusion of participants who die within 4 years of baseline testing—if a terminal decline in cognitive ability means that you are more likely to be closer to death, then perhaps you are simply also more likely to be closer to death if you are a smoker. In addition to failing to reach significance, the effect size for the relationship is reduced. Our final set of analyses included all of the main variables considered in this article, and smoking status did not reach significance in either. This is probably because of the inclusion of the fitness variable and the likely link between smoking and fitness.

We found that a 134.8 ng/L (1 *SD*) decrease in serum vitamin B-12 level at age 79 was associated with lower IQ scores at the same age. This relationship is in line with that found in the original article. Our results provide weight to the evidence for this relationship that exists within the literature, which is of particular importance given the conflicting evidence for this association (O’Leary et al., 2012). Although the effect size is relatively unchanged after the exclusion of those participants dying within 4 years of enrolment, the association between vitamin B-12 and cognition is no longer significant. Reduced dietary intake or reduced absorption can contribute to lower levels of serum vitamin B-12 and those who are unwell are therefore at an increased risk. It may be therefore, that cognitive function and vitamin B-12 both decline toward death and are not truly associated.

We must consider whether the consistency between the results when dementia cases were included and when cases were excluded is related to the possibility that the factors examined in this article are no longer associated with an increased risk for dementia after age 79. There is evidence to support this within the literature, with studies reporting no association between risk factors (vitamin-B-12, smoking, physical fitness) and dementia (Crystal et al., 1994; Piguet et al., 2003; Verghese et al., 2003; Wang et al., 1999), or at least a declining strength of association (*APOE* ε4; Corrada, Paganini-Hill, Berlau, & Kawas, 2013; Farrer et al., 1997). These findings have not, however, been comprehensively reinforced, and as a result, there is no widely accepted risk factor profile for dementia in the oldest-old. This likely relates to the difficulties in recruiting healthy persons in oldest age, and the potential for high rates of attrition because of morbidity and mortality in such cohorts (Sumic, Michael, Carlson, Howieson, & Kaye, 2007). Until there is a sufficient body of evidence disputing any risk factor for dementia in the oldest-old that is an accepted risk factor for dementia in earlier old-age, studies must continue to evaluate the effect of preclinical dementia in studies of nonpathological cognitive ageing.

We acknowledge the possibility that terminal decline has influenced our findings for vitamin B-12, smoking and *APOE* ε4, but we consider these results with caution given the reduced sample sizes (*n* = 309–315) for each of these analyses. The statistical power was reduced after these exclusions, relative to the original analyses, meaning that we were less likely to detect any association. We note that for physical fitness—the only finding that remained significant in these analyses—the reduction in power (relative to the complete sample) was only 6%, compared with a reduction in power of between 18 and 28% for the other analyses—none of which demonstrated a significant association. Furthermore, excluding a group likely to be experiencing cognitive decline reduces the amount of decline in remaining participants. If there is less variability in cognitive decline among those who remain in the sample, the likelihood of identifying factors associated with cognitive decline is diminished. Larger studies are required to reduce the impact of such an effect. A potentially effective way of reducing this effect when selecting dementia cases would be to identify probable cases of incipient dementia using ante-mortem neuroimaging data or postmortem neuropathological data. This could, however, lead to the potential misclassification of some participants, because pathological features of Alzheimer’s disease have been found in persons who died without cognitive impairment (Savva et al., 2009). Given the possible impact of terminal decline, we would suggest that studies investigating risk factors for cognitive decline in older age account for death occurring after 4 years or less within their analyses.

Fitness was found to be significantly associated with age 79 IQ in this article and the original article (Deary et al., 2006). After the exclusion of those participants who died within 4 years of testing, this was the only relationship that remained significant (*p* < .001). These results are in line with previous studies that have identified a relationship between increased fitness or exercise and decreased cognitive decline (Fitzpatrick et al., 2007; Taniguchi, Yoshida, Fujiwara, Motohashi, & Shinkai, 2012; Wendell et al., 2014). The results of our four main analyses go further than simply reinforcing previous findings—they add some validation to those studies without dementia follow-up (Bretsky et al., 2003; Fillenbaum et al., 2001; Sabia et al., 2012; Taniguchi et al., 2012). Given that the lack of follow-up for dementia ascertainment is so often a major criticism of studies investigating normal cognitive ageing, our findings are valuable in demonstrating the minimal impact of incident dementia in these follow-up analyses.

### Strengths and Limitations

The accuracy of the findings is limited by the possibility that participants who might otherwise have developed dementia could have died from other causes before the onset of dementia. By definition—as a result of excluding those who later developed dementia—fewer participants were included in each analysis than in previous studies. With the exception of not having knowledge of incipient dementia, the limitations present in the original studies persist. For example, we might expect the smokers in our cohort to be biased to being particularly fit, given that by age 80 they were relatively unaffected by serious smoking-related illness or death. We did not have sufficiently frequent cognitive assessments to investigate terminal decline fully and we, therefore, simply omitted those participants who died within 4 years of cognitive assessment at age 79 years. We cannot be confident in our findings relating to the effect of terminal decline because of reduced sample size, but given the possibility of an effect, we recommend that future studies account for death in their analyses. Our dementia ascertainment procedure did not include neuropathological examination after death. Whereas we could not exclude the presence of pathological findings typically associated with dementia syndromes, current evidence has shown that such findings are frequently observed within the brain tissues of older persons who died without cognitive impairment (Savva et al., 2009). Such findings demonstrate that, whereas pathological findings can confirm the etiology of dementia, their presence alone does not necessarily equate to the presence of a clinical dementia syndrome. This study aimed to determine the presence of the clinical syndrome of dementia; we would not, therefore, have expected this limitation to affect our results. As described in an earlier study (Sibbett et al., 2017b), our robust dementia ascertainment procedure included evidence gathered from multiple sources of data—including clinical assessment—and, as such, we were able to limit the number of potential missed cases. There will, however, always be limitations in the accuracy of such a methodology (Sibbett, Russ, Deary, & Starr, 2017a), primarily because the quality and quantity of available data will vary between participants. As such, we cannot entirely exclude the possibility that using a procedure with optimized sensitivity to identify cases may have found further cases and had an effect on the findings. A significant strength of our study is that we were able to include age 11 IQ in all analyses—both original and new—and so protect from the possibility of reverse causation by the influence of childhood IQ; in effect, we were able to have near-lifetime cognitive change as the outcome variable in these analyses. Using the LBC1921 as our study population also has a number of other benefits. Possible confounding effects are limited because of their ethnically, culturally, and geographically homogeneous nature, general good health, and narrow-age of the cohort. We recognize, though, that this also limits generalizability.

## Conclusions

These sensitivity analyses verify previous findings and demonstrate that preclinical or prodromal dementia had little influence on five LBC1921 studies that examined influences on nonpathological cognitive ageing. The presence of an *APOE* ε4 allele, smoking, lower physical fitness and lower vitamin B-12 were all associated with greater relative nonpathological lifetime cognitive decline. These findings allow us to suggest that the impact of incipient dementia would be minimal in studies with a similar methodology of excluding from analyses participants who self-report a diagnosis of dementia and/or score below an appropriate cut-off on a brief cognitive screening test.

## Supplementary Material

10.1037/pag0000241.supp

## Figures and Tables

**Table 1 tbl1:** Consensus Criteria for Dementia Case Ascertainment (Adapted from Sibbett et al., 2017b)

ANY of the following (without opposing evidence from same/other source)
Probable dementia
Dementia diagnosis on death certificate (any part)
Dementia diagnosed on clinical review (ICD-10/*DSM-IV*)
Dementia diagnosis in electronic general medical records (Trak)
Dementia diagnosis in electronic psychiatric records (PIMS)
ICD-10 criteria for dementia diagnosis met by data within any existing records
Possible dementia
Recorded cognitive impairment on death certificate
Cognitive impairment/decline recorded in notes, but incomplete evidence to meet ICD-10 diagnostic criteria
Possibility of dementia recorded in notes but no formal diagnosis/incomplete evidence to meet ICD-10 diagnostic criteria
*Note.* ICD-10 = International Statistical Classification of Diseases and Related Health Problems-Tenth Revision; *DSM-IV* = *Diagnostic and Statistical Manual for Mental Disorders-Fourth Edition*; Trak = TrakCare; PIMS = Patient Information Management System.

**Table 2 tbl2:** Main Findings From the Sensitivity Analyses and Terminal Decline Analyses, Compared With Findings From the Original Articles

		Main sensitivity analyses results			Original report results^a–e^			Terminal decline analyses results^g^		
Exposure	Outcome	Test statistic	Significance	Effect size (η_p_^2^)	Test statistic	Significance	Effect size (η_p_^2^)	Test statistic	Significance	Effect size (η_p_^2^)
*APOE* ε4 + (Article 1)	Age 79 MHT^f^ score	*F*(1, 357) = 4.27	.04	.012	*F*(1, 461) = 5.2^a^	.02^a^	.011^a^	*F*(1, 306) = 1.30	.255	.004
*APOE* ε4 + (Article 2)	Logical Memory Test Score- age 79	*F*(1, 355) = 8.16	.005	.022	*F*(1, 457) = 7.84^b^	.005^b^	.017^b^	*F*(1, 304) = 2.95	.087	.010
Smoking	Age 79 IQ	*F*(2, 360) = 3.67	.026	.020	*F*(2, 463) = 3.3^c^	.039^c^	.014^c^	*F*(2, 308) = 1.97	.141	.013
Vitamin B-12	Age 79 IQ	*S*β = .11	.014	—	*S*β = .095^d^	.011^d^	—	*S*β = .087	.075	—
Fitness^h^	Age 79 IQ	*S*β = .21	<.001	—	*S*β = .170^e^	<.001^e^	—	*S*β = .203	<.001	—
^a^ Deary et al. (2002). ^b^ Deary et al. (2004). ^c^ Deary et al. (2003). ^d^ Starr, Pattie, Whiteman, Deary, and Whalley (2005). ^e^ Deary, Whalley, Batty, and Starr (2006). ^f^ Moray House Test. ^g^ Main analyses excluding participants who died within 4 years of baseline testing. ^h^ Fitness general component formed using principal component analysis of sex- and height- adjusted 6-metre walk time, grip strength, and FEV_1_.										

**Table 3 tbl3:** Estimated Marginal Means and Effect Sizes for Categorical Risk Factors

			Dementia included		Dementia excluded		Dementia and death within 4 years excluded	
Exposure	Outcome test	Grouping	Estimated marginal mean (95% CI)	Effect size Cohen’s *d* (95% CI)	Estimated marginal mean (95% CI)	Effect size Cohen’s *d* (95% CI)	Estimated marginal mean (95% CI)	Effect size Cohen’s *d* (95% CI)
*APOE* ε4 (Article 1)	Age 79 MHT Score	*APOE ε*4 carrier	98.28 [96.24, 100.33]		98.86 [96.38, 101.34]		100.53 [97.95, 103.12]	
		*APOE* ε4 noncarrier	100.93 [99.74, 102.11]	−.23 [−.44, −.03]	101.82 [100.50, 103.14]	−.26 [−.51, −.01]	102.23 [100.87, 103.58]	−.16 [−.43, .12]
*APOE* ε4 (Article 2)	Verbal Fluency	*APOE ε*4 carrier	39.95 [37.86, 42.04]		40.68 [38.04, 43.32]		41.45 [38.56, 44.33]	
		*APOE* ε4 noncarrier	39.98 [38.73, 41.22]	.00 [−.21, .20]	40.02 [38.61, 41.43]	.06 [−.20, .31]	40.18 [38.67, 41.69]	.11 [−.17, .38]
	Raven’s Matrices	*APOE ε*4 carrier	30.73 [29.37, 32.09]		30.86 [29.22, 35.51]		31.52 [29.73, 33.32]	
		*APOE* ε4 noncarrier	31.77 [30.96, 32.58]	−.14 [−.34, .07]	32.65 [31.77, 33.53]	−.24 [−.49, .01]	32.80 [31.86, 33.74]	−.17 [−.45, .10]
	Logical Memory	*APOE ε*4 carrier	28.67 [26.49, 30.84]		29.38 [26.67, 32.08]		30.93 [27.96, 33.90]	
		*APOE* ε4 noncarrier	32.93 [31.64, 34.23]	−.35 [−.56, −.14]	33.82 [32.37, 35.26]	−.36 [−.61, −.11]	34.02 [32.47, 35.58]	−.25 [−.52, .02]
Smoking	Age 79 IQ	Current Smoker	95.80 [92.00, 99.59]		96.28 [92.25, 100.32]		98.15 [94.06, 102.24]	
		Ex-smoker	100.73 [99.31, 102.16]	−.44 [−.8, −.08]	101.27 [99.69, 102.84]	−.45 [−.84, −.06]	101.96 [100.28, 103.64]	−.36 [−.78, .06]
		Never-smoker^a^	100.92 [99.28, 102.56]	−.43 [−.79, −.06]	102.43 [100.53, 104.32]	−.53 [−.92, −.13]	102.68 [100.82, 104.54]	−.41 [−.83, .01]
*Note.* CI = confidence interval.								
^a^ Never smoking compared with current smoking.								
