# Supplementary material for: Does Incipient Dementia Explain Normal Cognitive Decline Determinants? Lothian Birth Cohort 1921
Source: Psychol Aging. 2018 May 10;33(4):674–84. doi: 10.1037/pag0000241 (PMC6001941; doi:10.1037/pag0000241)
Supplement: Supplementary file 1 [file z2m-3127_PAG-2017-1172_IntSup.zip › z2m999173127so1.docx]

**Supplemental Materials**

**Does Incipient Dementia Explain Normal Cognitive Decline Determinants? Lothian Birth Cohort 1921**

**by R. A. Sibbett et al., 2018, *Psychology and Aging***

**http://dx.doi.org/10.1037/pag0000241**

**Supplementary File A: Supplementary Tables**

*Note:* This supplementary file contains seven tables that display the complete results for each main analysis, as described in the main text.

Table S1. *Main Analysis and Terminal Decline Analysis Results for APOE ɛ4 (paper 1)*

|  | **Main Sensitivity Results** | | | **Terminal Decline Results** | | |
| --- | --- | --- | --- | --- | --- | --- |
|  | **F** | **Significance** | **Effect Size**  **(*ƞ_p_^2^)*** | **F** | **Significance** | **Effect Size**  **(*ƞ_p_^2^)*** |
| **Age 11 IQ** | 239.4 | <0.001 | 0.40 | 200.47 | <0.001 | 0.396 |
| **Sex** | 4.76 | 0.03 | 0.013 | 2.63 | 0.106 | 0.009 |
| ***APOE* ɛ4** | 4.27 | 0.039 | 0.012 | 1.30 | 0.255 | 0.004 |
| ***APOE* ɛ4*Sex** | 0.001 | 0.98 | <0.001 | 0.417 | 0.519 | 0.001 |

Table S2. *Main Analysis and Terminal Decline Analysis Results for APOE ɛ4 (Paper 2)*

|  |  | **Main Sensitivity Results** | | | **Terminal Decline Results** | | |
| --- | --- | --- | --- | --- | --- | --- | --- |
|  | **Dependent Variable** | **F** | **Significance** | **Effect Size (*ƞ_p_^2^)*** | **F** | **Significance** | **Effect Size (*ƞ_p_^2^)*** |
| ***APOE* ɛ4 Carrier** | Verbal Fluency score- age 79 | 0.19 | 0.664 | 0.001 | 0.59 | 0.443 | 0.002 |
|  | Ravens Matrices score- age 79 | 3.57 | 0.060 | 0.010 | 1.89 | 0.170 | 0.006 |
|  | Logical Memory score- age 79 | 8.16 | 0.005 | 0.022 | 2.95 | 0.087 | 0.010 |
| **Sex** | Verbal Fluency score- age 79 | 0.52 | 0.470 | 0.001 | 0.04 | 0.845 | <0.001 |
|  | Ravens Matrices score- age 79 | 10.92 | 0.001 | 0.030 | 2.41 | 0.122 | 0.008 |
|  | Logical Memory score- age 79 | 1.23 | 0.268 | 0.003 | 3.20 | 0.075 | 0.010 |
| **Age 11 IQ** | Verbal Fluency score- age 79 | 26.49 | <0.001 | 0.069 | 21.62 | <0.001 | 0.066 |
|  | Ravens Matrices score- age 79 | 85.49 | <0.001 | 0.194 | 69.31 | <0.001 | 0.186 |
|  | Logical Memory score- age 79 | 30.38 | <0.001 | 0.079 | 24.95 | <0.001 | 0.076 |

Table S3. *Main Analysis and Terminal Decline Analysis Results for Smoking*

|  | **Main Sensitivity Results** | | | **Terminal Decline Results** | | |
| --- | --- | --- | --- | --- | --- | --- |
|  | **F** | **Significance** | **Effect Size**  **(*ƞ_p_^2^)*** | **F** | **Significance** | **Effect Size**  **(*ƞ_p_^2^)*** |
| **Age 11 IQ** | 231.09 | <0.001 | 0.391 | 191.49 | <0.001 | 0.383 |
| **Sex** | 3.12 | 0.078 | 0.009 | 1.89 | 0.170 | 0.006 |
| **Smoker** | 3.67 | 0.026 | 0.020 | 1.97 | 0.141 | 0.013 |
| **Smoker*Sex** | 1.38 | 0.252 | 0.008 | 1.46 | 0.235 | 0.009 |

Table S4. *Main Analysis and Terminal Decline Analysis Results for Vitamin B-12*

|  |  | **Main Sensitivity Results** | | | | **Terminal Decline Results** | | | |
| --- | --- | --- | --- | --- | --- | --- | --- | --- | --- |
| **Model** | **Variable** | **Beta** | **Beta SE** | **Standardised Beta** | **Significance** | **Beta** | **Beta SE** | **Standardised Beta** | **Significance** |
| **Model 1** | Age 11 IQ | 0.585 | 0.045 | 0.584 | <0.001 | - | - | - | - |
|  | B-12* | 1.771 | 0.642 | 0.124 | 0.006 | - | - | - | - |
| **Model 2** | Age 11 IQ | 0.612 | 0.045 | 0.611 | <0.001 | 0.569 | 0.046 | 0.609 | <0.001 |
|  | Sex | -3.257 | 1.272 | -0.115 | 0.011 | -2.825 | 1.285 | -0.108 | 0.029 |
|  | *APOE* ɛ4 status | 1.884 | 1.505 | 0.056 | 0.211 | 0.394 | 1.553 | 0.012 | 0.800 |
|  | Smoking status | 1.428 | 0.995 | 0.065 | 0.152 | 1.220 | 1.022 | 0.059 | 0.234 |
|  | Statin use | 4.238 | 2.517 | 0.080 | 0.093 | 3.513 | 2.599 | 0.070 | 0.178 |
|  | Total number of drugs | -0.947 | 0.281 | -0.159 | 0.001 | -1.072 | 0.284 | -0.194 | <0.001 |
|  | B-12* | 1.535 | 0.624 | 0.110 | 0.014 | 1.127 | 0.631 | 0.087 | 0.075 |

*Note.* *Standardised as z-scores

Table S5. *Main Analysis and Terminal Decline Analysis Results for Physical Fitness*

|  | **Main Sensitivity Results** | | | | | **Terminal Decline Results** | | | | |
| --- | --- | --- | --- | --- | --- | --- | --- | --- | --- | --- |
|  | **R^2^ change** | **Beta** | **Beta SE** | **Standardised Beta** | **Significance** | **R^2^ change** | **Beta** | **Beta SE** | **Standardised Beta** | **Significance** |
| **Age 11 IQ** | 0.383 | 0.549 | 0.042 | 0.559 | <0.001 | 0.385 | 0.534 | 0.044 | 0.558 | <0.001 |
| **Fitness trait** | 0.047 | 2.976 | 0.559 | 0.209 | <0.001 | 0.048 | 2.851 | 0.602 | 0.203 | <0.001 |
| **Social class** | 0.18 | -2.285 | 0.726 | -0.136 | 0.002 | 0.013 | -1.913 | 0.763 | -0.116 | 0.013 |
| **Sex** | 0.08 | -2.535 | 1.135 | -0.088 | 0.026 | 0.007 | -2.335 | 1.170 | -0.085 | 0.047 |

*Note.* Smoking status and *APOE* ɛ4 carrier status did not enter the model (*p*>0.05).

Table S6. *Multivariable Linear Regression Results: All main variables and Age 79 IQ Scores*

|  | **R^2^ change** | **Beta** | **Beta SE** | **Standardised Beta** | **Significance** |
| --- | --- | --- | --- | --- | --- |
| **Age 11 IQ** | 0.342 | 0.562 | 0.043 | 0.563 | <0.001 |
| **Fitness trait** | 0.058 | 3.658 | 0.620 | 0.255 | <0.001 |
| **Vitamin B-12*** | 0.021 | 2.507 | 0.735 | 0.147 | 0.001 |
| **Sex** | 0.013 | -3.284 | 1.226 | -0.115 | 0.008 |

*Note.* *Vitamin B-12 standardised as z-scores. Smoking status and *APOE* ɛ4 carrier status did not enter the model (p>0.05).

Table S7. *Multivariate General Linear Modelling Results: All Main Variables and Verbal Fluency, Raven’s Matrices and Logical Memory Test Scores*

|  | **Dependent Variable** | **F** | **Significance** | **Partial Eta Squared** |
| --- | --- | --- | --- | --- |
| ***APOE* ɛ4 Status** | Verbal Fluency score- age 79 | 0.44 | 0.506 | 0.001 |
|  | Ravens Matrices score – age 79 | 1.85 | 0.175 | 0.006 |
|  | Logical Memory score- age 79 | 5.47 | 0.020 | 0.018 |
| **Sex** | Verbal Fluency score- age 79 | 0.32 | 0.575 | 0.001 |
|  | Ravens Matrices score – age 79 | 13.27 | <0.001 | 0.042 |
|  | Logical Memory score- age 79 | 1.15 | 0.284 | 0.004 |
| **Age 11 IQ** | Verbal Fluency score- age 79 | 18.96 | <0.001 | 0.059 |
|  | Ravens Matrices score – age 79 | 66.79 | <0.001 | 0.182 |
|  | Logical Memory score- age 79 | 21.35 | <0.001 | 0.066 |
| **Smoking Status** | Verbal Fluency score- age 79 | 0.50 | 0.609 | 0.003 |
|  | Ravens Matrices score – age 79 | 0.84 | 0.432 | 0.006 |
|  | Logical Memory score- age 79 | 0.48 | 0.617 | 0.003 |
| **Vitamin B-12*** | Verbal Fluency score- age 79 | 0.38 | 0.539 | 0.001 |
|  | Ravens Matrices score – age 79 | 3.82 | 0.052 | 0.013 |
|  | Logical Memory score- age 79 | 0.878 | 0.349 | 0.003 |
| **Fitness** | Verbal Fluency score- age 79 | 12.28 | 0.001 | 0.039 |
|  | Ravens Matrices score – age 79 | 17.98 | <0.001 | 0.056 |
|  | Logical Memory score- age 79 | 0.24 | 0.627 | 0.001 |

*Note.* *Vitamin B-12 standardised as z-scores
