# Supplementary material for: Does Incipient Dementia Explain Normal Cognitive Decline Determinants? Lothian Birth Cohort 1921
Source: Psychol Aging. 2018 May 10;33(4):674–84. doi: 10.1037/pag0000241 (PMC6001941; doi:10.1037/pag0000241)
Supplement: Supplementary file 1 [file z2m-3127_PAG-2017-1172_IntSup.zip › z2m999173127so2.docx]

**Supplemental Materials**

**Does Incipient Dementia Explain Normal Cognitive Decline Determinants? Lothian Birth Cohort 1921**

**by R. A. Sibbett et al., 2018, *Psychology and Aging***

**http://dx.doi.org/10.1037/pag0000241**

**Supplementary File B: Risk Factor and Dementia Interactions**

Following each of the main individual analyses, we completed a subsequent analysis in which participants with probable dementia or no dementia were included. We excluded participants with possible dementia. Forming a between-groups variable (dementia or no dementia), we included an interaction term with the risk factor (e.g. dementia status*smoking status) to determine whether the effect of the risk factor varied as a function of the dementia group.

*APOE ɛ4 Paper 1: Moray House Test (MHT) score as outcome.(I. J. Deary et al., 2002)*

When we included those participants without dementia (n=363) and those with probable dementia (n=101)—a future diagnosis of probable dementia was associated with a lower standardised MHT score at age 79 (F_1,456_=8.13, *p*=0.005, *ƞ_p_^2^*=0.018). The *APOE* ɛ4 carrier status by dementia status interaction was not significant (F_1,456_=1.10, *p*=0.30, *ƞ_p_^2^*=0.002), demonstrating that the effect of *APOE* ɛ4 was not significantly different between the two dementia groups. The mean MHT score (standardised) at age 79 (95% CI) for *APOE* ɛ4 carriers was 96.47 (92.88, 100.06) for those with dementia and 98.89 (96.43, 101.35) for those without dementia. The mean MHT score at age 79 for *APOE* ɛ4 non-carriers was 96.48 (93.70, 99.27) for those with dementia and 101.73 (100.43, 103.03) for those without dementia.

*APOE ɛ4 Paper 2: Logical Memory, Raven’s Matrices, and verbal fluency as outcomes.(I. Deary, Whiteman, Pattie, & Starr, 2004)*

When the analysis was repeated with the inclusion of probable dementia cases, a future diagnosis of dementia was found to contribute to Raven’s Matrices (F_1,454_= 8.24, p=0.004, ƞ_p_^2^ =0.018) and Logical Memory test scores (F_1,454_= 5.65, p=0.018, ƞ_p_^2^ =0.012) at age 79. The *APOE* ɛ4 carrier status by dementia status interaction was associated with Raven’s Matrices test score at age 79 (F_1,454_= 7.75, p=0.006, ƞ_p_^2^ =0.017) but not Logical Memory (F_1,454_=0.49, p=0.484, ƞ_p_^2^ =0.001) or Verbal Fluency test scores (F_1,454_= 0.20, p=0.66, ƞ_p_^2^=<0.001). The effect of *APOE* ɛ4 on Raven’s Matrices test score was therefore different between those who developed dementia and those who did not, but no difference was noted for Logical Memory or Verbal Fluency. The mean (95% CI) Raven’s Matrices test score for *APOE* ɛ4 carriers was 30.84 (29.19, 32.49) for those without dementia and 30.79 (28.45, 33.12) for those who developed dementia. For noncarriers, the mean test score was 32.65 (31.77, 33.53) for those without dementia and 27.58 (25.71, 29.45) for those who developed dementia.

*Smoking*

Repeating the main analysis with the inclusion of participants who developed probable dementia (n=100) and participants who remained dementia free (n=367), we found that dementia was associated with age 79 IQ (F_1,457_=6.60, *p*=0.01, *ƞ_p_^2^* =0.014). The smoking status by dementia status interaction was not associated with age 79 IQ (F_2,457_=0.513, *p*=0.599, *ƞ_p_^2^* =0.002). The effect of smoking was not therefore significantly different between the dementia groups. For those without dementia, the estimated marginal mean scores (95% CI) were 96.25 (92.24, 100.25) for current smokers, 101.28 (99.72, 102.85) for ex-smokers and 102.29 (100.43, 104.14) for never-smokers. For those who developed probable dementia, the mean scores were 87.40 (74.87, 99.24) for current smokers, 97.61 (94.28, 100.93) for ex-smokers and 96.53 (93.51, 99.54) for never-smokers.

*Vitamin B-12*

When we include only those participants with probable dementia (n=101) or no dementia (n=367), there was no significant association between the interaction term (dementia and vitamin B-12) and age 79 IQ (*Sβ*=-0.030, *p*=0.463). Future probable dementia was shown to be associated with age 79 IQ (*Sβ*=-0.141, *p*<0.001).

*Physical Fitness*

Including in the analysis only those with probable dementia (n=97) or no dementia (n=359) – the interaction variable between dementia and fitness did not enter the model (*p*>0.05). Future probable dementia was again associated with age 79 IQ (*Sβ*=-0.14, *p*<0.001, *R^2^*=0.021).

Deary, I., Whiteman, M., Pattie, A., & Starr, J. (2004). Apolipoprotein E gene variability and cognitive functions at age 79: A follow-up of the Scottish mental survey of 1932. *Psychology and Aging, 19*(2), 367-371.

Deary, I. J., Whiteman, M. C., Pattie, A., Starr, J. M., Hayward, C., Wright, A. F., . . . Whalley, L. J. (2002). Ageing: Cognitive change and the APOE [epsiv]4 allele. *Nature, 418*(6901), 932-932.
